# Investigating the associations between productive housework activities, sleep hours and self-reported health among elderly men and women in western industrialised countries

**DOI:** 10.1186/s12889-017-4979-z

**Published:** 2018-01-11

**Authors:** Nicholas Kofi Adjei, Tilman Brand

**Affiliations:** 1 Department of Prevention and Evaluation, Leibniz Institute for Prevention Research and Epidemiology - BIPS, Germany, Unit Social Epidemiology, Achterstrasse 30, D-28359 Bremen, Germany; 2 Health Sciences Bremen, University of Bremen, Bremen, Germany

**Keywords:** Self-reported health, Gender, Elderly, Sleep duration, Housework activities

## Abstract

**Background:**

After retirement, elderly men and women allocate more time to housework activities, compared to working-age adults. Nonetheless, sleep constitutes the lengthiest time use activity among the elderly, but there has not been any study on the associations between time spent on housework activities, sleep duration and self-reported health among the older population. This study not only examined individual associations between self-reported health and both housework activities and sleep duration, but it also explored self-reported health by the interaction effect between housework activities and sleep duration separately for men and women.

**Methods:**

Pooled data from the Multinational Time Use Study (MTUS) on 15,333 men and 20,907 women from Germany, Italy, Spain, UK, France, the Netherlands and the US were analysed. Multiple binary logistic regression models were used to examine the associations between three broad categories of housework activities ((1) cooking, cleaning and shopping, (2) gardening and maintenance; (3) childcare) and health. We further investigated the extent to which total housework hours and sleep duration were associated with self-reported health for men and women separately.

**Results:**

We found a positive association between time devoted to housework activities, total housework and health status among elderly men and women. Compared to those who spent 1 to 3 h on total productive housework, elderly people who spent >3 to 6 h/day had higher odds of reporting good health (OR = 1.25; 95% CI = 1.14–1.37 among men and OR = 1.10; 95% CI = 1.01–1.20 among women). Both short (<7 h) and long (>8 h) sleep duration were negatively associated with health for both genders. However, the interactive associations between total productive housework, sleep duration, and self-reported health varied among men and women. Among women, long hours of housework combined with either short or long sleep was negatively associated with health.

**Conclusions:**

Although time allocation to housework activities may be beneficial to the health among both genders, elderly women have higher odds of reporting poor health when more time is devoted total housework combined with either short or long sleep duration.

**Electronic supplementary material:**

The online version of this article (10.1186/s12889-017-4979-z) contains supplementary material, which is available to authorized users.

## Background

Due to the increase in life expectancy among older adults, time spent in retirement has increased remarkably [1]. In fact, the retirement of an individual affects the distribution of allocated time to the various task of life. A plethora of literature has examined how elderly men and women spend their time after retirement. Most of the related literature that examined post-retirement time use showed that elderly men and women are more involved in social roles and activities such as voluntary work [2–4], leisure activities [5, 6], grandparenting [7, 8] and household activities [6, 9], than their younger counterparts [9–11].

Household activities are part of the daily lives of older people. These activities have become their main “productive work” after retirement [12]. Using longitudinal data, Szinovacz [13] found that retirees devoted more time to housework activities than their working spouses. However, evidence suggests that gender inequality in the division of household labor largely persists in high-income societies even after retirement [13, 14]. Most studies confirm that elderly women spend more time on housework activities than men [14–16], although time allocated to housework activities among men has increased over recent years [15]. Again, women typically perform routine, repetitive tasks such washing clothes, cooking and cleaning house [17, 18], while men are responsible for occasional tasks such as household repair works, vehicle maintenance and yard work [17, 18]. It has been suggested that this inequitable division of housework is one of the factors that contribute to the observed adverse health differences among men and women including psychological distress [19] and depressive symptoms [20]. These adverse health outcomes are stronger for women than men in their prime working age [21]. Thus, gendered work-life imbalance could be a contributing factor for health inequalities, where women are more likely than men to report poorer health [19]. Although the gendered distribution of housework seems to be correlated with low psychological well-being and poor health among women, there is some evidence that suggests housework may have a positive impact on physical health among elderly men and women [14, 22].

Nonetheless, an important aspect of daily time use activities among older men and women is sleep. Sleep is one of the most important determinants of health [23]. Thus, the duration and quality of sleep have significant health consequences for children, adults and the elderly. Among the elderly, sleep constitutes the lengthiest daily activity [24]. This is expected because the increasing prevalence of health conditions at older age restricts time allocation for other daily activities [6]. Time devoted to sleep is therefore crucial because it has been shown to be correlated with health among older adults. Among adults, short sleep has been found to be associated with some adverse health outcomes including cardiovascular disease and obesity [25]. Conversely, long sleep duration has been linked with increased risk of mortality and morbidity [23].

Time resources are limited within 24 h or 1440 min in a day. Therefore, within this period, time devoted to a particular activity influences time allocation to other daily activities. For example, more time allocation to housework may decrease the amount of time devoted to sleep or other time use activities such as leisure.

To our knowledge, only one study has examined the effect of various housework activities on health among older adults [26]. Moreover, the gender-specific interactive effects of time spent on housework and sleep duration on self-reported health have yet to be investigated among the elderly. Accordingly, this study seeks to explore the relationship between housework activities, total housework and sleep on self-reported health among the elderly in high-income countries. The following two questions will be addressed:

1. How does time spent on housework activities impact the health among the elderly? To what extent do these effects vary by gender?
2. To what extent is the association between housework activities and health moderated by sleep hours among men and women?

## Methods

### Data

Data came from the Multinational Time Use Study (MTUS, version W53), a cross-national harmonized and comparative time-use database. The Centre for Time Use Research at the University of Oxford organized and collated this data, which was a collection of national randomly-sampled time-use surveys conducted by institutions in 25 countries [27]. This data set contains information on time allocation to various daily tasks, as well as socio-economic and demographic background information of the respective diarist. Diaries were self-administered, followed by a personal visit in most countries. In the interviews, individual participants reported the total time spent per day on 41 activities in 5, 10 or 15-min intervals [27], during a randomly assigned day in a week in Spain, Italy, Germany France and the US or two days (weekday and weekend) in the UK. In the Netherlands, individuals report their activities throughout the 24 h of the day for seven consecutive days. We excluded individuals whose diaries did not sum up to 1440 min (24 h) of activities during the day. Only primary or “main” activities were included in this analysis. Information on secondary activities (activities done while multitasking) were not captured. After considering the minimum retirement age in most EU countries., we limited our sample to individuals who were 65 years and above. For the selection of countries, we selected countries that incorporate health and wellbeing measures into their diary collections. The countries used for the final analysis after these exclusions were the United Kingdom (survey year: 2000; response rate: 45%), the United States (survey year: 2003, response rate: 57%), Spain (survey year: 2002; response rate: 64%), Italy (survey year: 2002; response rate: 92%), Germany (survey year: 2001; response rate: 96%), France (survey year: 1998, response rate: 88%) and the Netherlands (survey year: 2000; response rate: 25%).

### Independent variables

Time spent on housework activities and sleep were the two independent variables used for this study. Sleep time and time allocated to housework activities were directly estimated from the data. Because it is not easy to identify productive household activities in time use research, we used the “third party” criterion to identify and select these activities. This approach is widely used in the literature in defining productive household activities [28]. By this criterion, “if an activity is of such a character that might be delegated to a paid worker, then that activity shall be deemed productive” [29]. In other words, this comprises housework that people can pay others to perform for them. Hence, activities such as cooking, gardening, washing, maintenance, laundry, grocery shopping and childcare were considered as productive housework activities. In this current study, we focused on three broad categories of housework activities, in line with Wen et al. [26]:

- cooking, cleaning and shopping
- gardening and maintenance
- childcare

Each category of time use activity was measured in minutes per day. Total housework hours were measured in hours per day. Sleep hours or duration was defined as the total amount of time devoted to sleep. This encompasses all forms of sleep (including daytime sleep and naps). Sleep hours were classified into three categories, i.e., <7 h (short sleep duration), 7–8 h (optimal sleep duration), and >8 h (long sleep duration), based on existing cut-offs in epidemiologic studies [30].

### Dependent variable

The dependent variable was self-reported health. In the time-use survey, the question posed to the diarist was “How is your health in general; would you say that it is …?” response options: zero (poor) to three (very good). We categorized the responses into poor (poor or fair) and good (good or very good) [14]. Self-reported health has been shown to be a reliable and accurate measure of current health status [31, 32].

### Covariates

Covariates included in the analyses were age, education (less than secondary education, completed secondary education and above secondary education), housing tenure (owner-occupier, renting), employment status (not working for pay, currently in paid employment), household size (1, 2, 3+) and car ownership (no car, one car and two or more cars).

### Statistical analysis

The first part of the analysis was primarily descriptive, where information on distributional characteristics of all variables including the mean time allocated to the various productive housework activities was provided. In the second part of the analysis, we applied binary logistic regression to model the association between self-reported health and each of the three broad housework categories, total housework and sleep hours. The multivariate regression models included other time use activities (paid work, active leisure, passive leisure and personal activities, see Additional file 1: Table S2)*.* In the third part, we examined the combined association of total productive housework hours and sleep duration (short sleep duration, optimal sleep duration and long sleep duration) on self-reported health. Here, we investigated twelve combinations as follows: four groups of total productive housework hours (housework <1 h/day, housework 1 to 3 h/day, housework >3 to 6 h/day, housework >6 h/day) × three groups of sleep hours (sleep <7 h/day, sleep >7 to 8 h/day, sleep >8 h/day). The >7 to 8 h/day sleep duration and 1 to 3 h/day time spent on productive housework categories were chosen as the reference. The analyses were done separately for men and women. All statistical analyses were performed in STATA version 14 [33].

## Results

### Descriptive statistics

The descriptive statistics for respondents stratified by gender are shown in Table 1.

**Table 1:** General description of the study sample (in percentages, means and SD), men and women

	Men (15,333)		Women (20,907)	
	Mean / %	SD	Mean / %	SD
Sociodemographic and economic factors				
*Age*	72.39	5.06	73.19	5.21
65–69	35.7%		31.0%	
70–74	28.3%		26.3%	
75–79	20.0%		20.7%	
80+	16.1%		22.0%	
*Education*				
Incomplete Sec. or less	49.6%		58.3%	
Secondary completed	30.1%		28.3%	
Tertiary Completed or above	20.3%		13.3%	
*Land tenure*				
Renting	22.5%		26.8%	
Owner occupier	77.5%		73.2%	
*Employment Status*				
Not working for pay	91.0%		95.3%	
Currently in paid employment	9.0%		4.7%	
*Household size*	2.20	1.02	1.89	1.08
1 member	18.2%		41.6%	
2 members	59.1%		41.3%	
3+ members	22.8%		17.1%	
Time use Activity				
*Cleaning, cooking & shopping mins/day*	84.74	97.02	217.88	139.45
0 min	27.4%		7.2%	
>0 to 60	27.4%		9.7%	
>60 to 120	18.2%		11.4%	
>120	26.9%		71.8%	
*Gardening and maintenance mins/day*	68.56	109.37	38.54	77.43
0 min	50.4%		59.2%	
>0 to 60	17.4%		21.4%	
>60 to 120	11.0%		9.3%	
>120	21.2%		10.2%	
*Childcare mins/day*	1.41	15.02	2.07	19.59
0 min	98.3%		97.7%	
>0 to 60	1.0%		1.2%	
>60 to 120	0.3%		0.5%	
>120	0.4%		0.5%	
*Total Housework hours/day*	3.09	2.65	4.72	2.71
Less than 1	26.4%		10.0%	
1 to 3	27.1%		16.4%	
>3 to 6	31.9%		42.8%	
>6	14.7%		30.8%	
*Sleep hours/day*	9.43	2.07	9.34	2.10
less than 7	6.7%		6.9%	
>7 to 8	17.4%		18.2%	
>8	75.9%		75.0%	

Women in the study were slightly older than men. The mean age of women was 73.2 years and for men 72.4 years. Men had higher educational attainment than women. About 20.3% of elderly men and 13.3% of women reported having a tertiary education. Elderly men were more likely to own a house than women (77.5% vs 73.2%). Regarding employment status, about 9.0% of older men were in paid employment, compared to 4.7% of women. The average number of people in the household was similar for both men and women (approximately 2 members).

Gender differences were also found in time allocation to productive housework activities. Older men and women both allocated more time to cleaning and cooking than to occasional task such as gardening and maintenance. However, men spent remarkably fewer hours on cleaning, cooking and shopping than women (88.7 min/day vs 217.9 min/day). On the other hand, women devoted fewer hours on gardening and maintenance than men (38.5 min/day vs 68.6 min/day). Regarding the time allocation to total housework, women devoted most hours to these activities (4.7 h per day) compared to men (3.1 h per day). A cross-country comparison in Additional file 2: Table S1 revealed that the most time spent on total housework among elderly women was found in Italy (5.2 h per day) and Germany (5.1 h per day), while the lowest value was observed in the US (4.0 h per day). In contrast, elderly men in Italy devoted the least time to total housework activities (2.7 h per day), while the most time spent on these activities was found in Germany (4.2 h per day).

Time allocated to sleep hours was similar in both genders. Elderly men and women slept for approximately 9 h per day (including daytime sleep and naps). Again, we observed that there were no differences in time devoted to sleep hours among men and women within-countries, but there were cross-national variations (Additional file 2: Table S1). For instance, elderly men and women in Spain and France devoted the most time to sleep hours (approximately 10 h per day), while the average time spent on these activities in the other countries was 1 h less.

### Logistic regression

The results of the adjusted OR and 95% CI for the association between the three broad productive housework activities, total housework, sleep hours and the outcome self-reported health are shown in Tables 2 and 3.

**Table 2:** Multivariate associations between good self-reported health status and housework, pooled data of 7 countries. Men, women 65+ years old

Variables	Men	Women
	aOR (95% CI)	aOR (95% CI)
Time use Activities		
*Cleaning, cooking & shopping mins/day*		
0 min (ref)	1.00 (reference)	1.00 (reference)
>0 to 60	1.23 (1.12–1.35)**	1.61 (1.38–1.89)**
>60 to 120	1.13 (1.02–1.26)*	1.46 (1.25–1.70)**
>120	1.58 (1.43–1.74)**	1.48 (1.30–1.68)**
*Gardening and maintenance mins/day*		
0 min (ref)	1.00 (reference)	1.00 (reference)
>0 to 60	1.27 (1.16–1.40)**	1.31(1.22–1.41)**
>60 to 120	1.40(1.25–1.56)**	1.43(1.29–1.59)**
>120	1.80(1.64–1.98)**	1.56(1.41–1.73)**
*Childcare mins/day*		
0 min (ref)	1.00 (reference)	1.00 (reference)
>0 to 60	1.18 (0.85–1.65)	1.11 (0.85–1.46)
>60 to 120	1.60 (0.85–3.00)	1.25 (0.83–1.87)
>120	1.81 (1.03–3.20)*	2.46 (1.63–3.72)**
Pseudo R2	0.0815	0.1049
Log Likelihood	−9728.2581	−12,635.574

Notes: aOR- adjusted Odd Ratio, ** p < 0.01, * p < 0.05. CI: confidence interval. Adjusted by age, education, householdsize, land tenure, employment status and other time use activities (active leisure, paid work, passive leisure & personal activities)

**Table 3:** Multivariate associations between good self-reported health status, total housework and sleep hours, pooled data of 7 countries. Men, women 65+ years old

Variables	Men	Women
	aOR (95% CI)	aOR (95% CI)
Total housework hours/day		
*less than 1*	0.74 (0.67–0.81)**	0.69 (0.60–0.78)**
1 to 3	1.00 (reference)	1.00 (reference)
>3 to 6	1.25 (1.14–1.37)**	1.10(1.01–1.20)**
>6	1.86 (1.65–2.11)**	1.38(1.24–1.53)**
Sleep hours/day		
*less than 7*	0.83 (0.71–0.97)*	0.84 (0.73–0.95)*
>7 to 8	1.00 (reference)	1.00 (reference)
>8	0.78 (0.71–0.85)**	0.75 (0.69–0.81)**
Pseudo R2	0.0834	0.1053
Log Likelihood	−9707.8736	−12,629.694

Notes: aOR- adjusted Odd Ratio, ** p < 0.01, * p < 0.05. CI: confidence interval. Adjusted by age, education, household size, land tenure, employment status and other time use activities (active leisure, paid work, passive leisure & personal activitie

Among men and women, we found a positive association between housework activities and self-reported health. However, there were differences in the magnitude of the associations. Time devoted to both routine and repetitive housework activities was significantly associated with good health. We observed that elderly people who spent more than 120 min/day on cleaning, cooking and shopping activities had higher odds of reporting good health (OR = 1.58; 95% CI = 1.43–1.74 among men and OR = 1.48; 95% CI = 1.30–1.68 among women) compared to those who devoted no time to these activities. Gardening and maintenance activities were associated with higher odds for good health. Older people who spent more than 120 min/day on these activities were more likely to report good health (OR = 1.80; 95% CI = 1.64–1.98 among men and OR = 1.56; 95% CI = 1.41–1.73 among women) compared to those who did not allocate any time to these activities. The odds of reporting good health were significantly higher (OR = 1.81; 95% CI = 1.03–3.20 among men and OR = 2.46; 95% CI = 1.63–3.72 among women) for elderly people who spent more than 120 min/day on childcare activities compared to those who devoted no time to childcare.

Furthermore, Table 3 shows that total productive housework activities and sleep duration were also related to health in both genders. In the model, we found a statistically significant association between good health and time devoted to housework. Elderly people who spent more than 6 h on housework activities had higher odds of reporting good health (OR = 1.86; 95% CI = 1.65–2.11 among men and OR = 1.38; 95% CI = 1.24–1.53 among women) compared to those who spent 1 to 3 h on these activities. The odds of reporting good health status were lower among older people who devoted less than 7 h (OR = 0.83; 95% CI = 0.71–0.97 among men and OR = 0.84; 95% CI = 0.73–0.95 among women) and more than 8 h (OR = 0.78; 95% CI = 0.71–0.85 among men and OR = 0.75; 95% CI = 0.69–0.81 among women) compared to those who reported a sleep duration between 7 to 8 h.

### Interactions

**Fig. 1:**
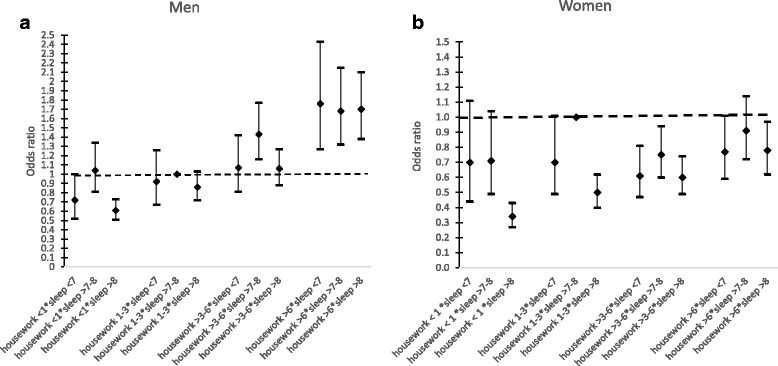
**a** Combined associations between good self-reported health status, total housework hours and sleep hours, pooled data of 7 countries. Men, 65+ years old. **b** Combined associations between good self-reported health status, total housework hours and sleep hours, pooled data of 7 countries. Women, 65+ years old

Table 4 and Fig. 1a and b shows the combined associations of total housework hours and sleep duration on self-reported health by gender.

**Table 4:** Combined associations between good self-reported health status, total housework hours and sleep hours, pooled data of 7 countries. Men, women 65+ years old

Combinations	Sleep <7 h/day	Sleep >7 to 8 h/day	Sleep >8 h/day
	aOR (95% CI)	aOR (95% CI)	aOR (95% CI)
Men			
Housework <1 h/day	0.72 (0.52–1.00)*	1.04 (0.81–1.34)	0.61 (0.51–0.73)**
Housework 1 to 3 h/day	0.92 (0.67–1.26)	1.00 (reference)	0.86 (0.72–1.03)
Housework >3 to 6 h/day	1.07 (0.81–1.42)	1.43 (1.16–1.77)**	1.06 (0.88–1.27)
Housework >6 h/day	1.76 (1.27–2.43)**	1.68 (1.32–2.15)**	1.70 (1.38–2.10)*
Women			
Housework <1 h/day	0.70 (0.44–1.11)	0.71 (0.49–1.04)*	0.34 (0.27–0.43)**
Housework 1 to 3 h/day	0.70 (0.49–1.01)*	1.00 (reference)	0.50 (0.40–0.62)**
Housework >3 to 6 h/day	0.61 (0.47–0.81)**	0.75 (0.60–0.94)*	0.60 (0.49–0.74)**
Housework >6 h/day	0.77 (0.59–1.01)*	0.91 (0.72–1.14)	0.78 (0.62–0.97)*

Notes: aOR- adjusted Odd Ratio, ** p < 0.01, * p < 0.05. CI: confidence intervals. Adjusted by age, education, household size, land tenure, employment status and other time use activities (active leisure, paid work, passive leisure & personal activities)

Among both genders, any combination of sleep duration not equal to 7–8 h and less than one hour spent on total housework was significantly associated with poorer health. On the other hand, the odds of reporting good health were significantly higher among older men who devoted more than 6 h/day of housework with any sleep hours category as compared to the reference group. Conversely, the odds of reporting good health were significantly lower among elderly women with any combination of sleep hours category with more hours of housework as compared to the reference group defined as above.

## Discussion

This study explored the individual association between housework activities, sleep duration and self-reported health, and additionally examined the combined associations of total housework and sleep duration on self-reported health among elderly men and women in selected high-income countries. As far as we know, this is the first study to analyze these interactive associations among elderly men and women in six European countries and the US using time use data. On the descriptive level, our study showed that elderly women allocate more time to routine and repetitive housework such as cleaning and cooking, whereas elderly men tend to devote more time to occasional tasks such as gardening and maintenance. Interestingly, both routine and occasional housework activities were positively associated with health among elderly men and women, but the magnitude of the association varied. Regarding time spent on total housework activities, there were gender and cross-country differences. Women spent more time than men in housework activities, consistent with previous literature [11, 12]. However, we observed a cross-country variation in time devoted to these activities. The result as shown in Additional file 2: Table S1 revealed that elderly women in the southern European countries and Germany allocated most time to total housework activities. In contrast, men’s total housework activities were about 2 h per day less than that of women in the southern European countries. Meanwhile, the difference in housework among older men and women was approximately 1 h per day in Germany. Gender differences in the allocation of time especially for housework may to some extent be explained by cultural and social norms [34]. These norms may shape total work distribution and time use patterns of the various task of life among men and women. For instance, in the southern European countries where gender roles are still shaped in a more traditional way [34], women devote a significant amount of time to housework activities while the amount of time devoted to other time use activities may be reduced.

### Housework activities, sleep hours and health

Although gender inequality in time allocation to housework activities exists [15, 16], we found that time devoted to the three broad categories of housework activities were positively associated with health among elderly women and men. Our findings of the association between routine housework activities (cleaning, cooking and shopping) and health contrast with a recent study conducted in China. Wen et al. [26] found no significant association of cooking, cleaning and grocery shopping and health among older men, but washing clothes and house cleaning were negatively associated with health among women. Meanwhile, our results corroborate a longitudinal study of 2761 older Americans aged 65 years [35]. Glass et al. [35] found productive housework activities such as cooking, shopping and gardening to be associated with lower risk of mortality. To these effects, we note that the three broad categories of housework activities involve some form of physical activities which may be beneficial to health among older adults [36]. Gardening and maintenance activities may increase fitness level and muscle strength because they require some form of physical exertion such as carrying equipment for repair works, lawn mowing, shoveling, digging holes and carrying soil. Previous studies have also stressed the health benefits of gardening for older adults; such benefits include physical health, psychological health, cognitive ability, and low risk of depression [37, 38]. Park et al. [39] recently reported that gardening has a positive effect on the blood lipid profiles, blood pressure and level of inflammatory markers in blood.

We also found a positive association between childcare and health status among older people. Although the amount of time devoted to childcare activities among the elderly is very small compared to young adults [6], caregiving support, especially caring for grandchildren, has been linked with good psychosocial health [40, 41]. Ku et al. [41] found that grandparenting was positively associated with good self-reported health and lower risk of depression. However, in some instances, more time devoted to childcare among older adults may have a negative impact on their physical or mental health [42], especially among custodial grandparents [43, 44].

Regarding time devoted to total housework hours, the result showed a positive association with health status among both genders. The few studies that examined the effects of total time spent on housework and health among elderly men have given inconsistent results [26, 35, 45, 46]. Lawlor et al. [46] reported that heavy housework was not associated with reduced likelihood of being overweight among British women aged 60 to 79 years.

Among the working population, unequal division of household labor has been linked with adverse health outcomes especially among women [19, 21]. One main hypothesis that has been advanced to explain these gender-specific inequalities in health is the “double burden” of work hypothesis. It has been postulated that the combination of paid market work and domestic work may be more stressful for women than men [47], which may affect women’s health negatively [19, 48]. Research findings [6, 34] indicate that the patterns and distribution of time use vary largely among the elderly and the working population. Therefore, the elderly may not have the same time constraints of combining both paid work and household activities like the working-age adults. In this case, the “double burden” of work hypothesis might lose its premise in explaining the effect of total housework on health among elderly men and women. In our view, housework activities by the older population can be perceived as domestic leisure activities [12] and forms of domestic physical activities [38] rather than “work overload”, given a changed time availability after retirement. Hence, there is overall evidence for a positive association between housework activities and health status among elderly men and women.

Regarding sleep duration and self-reported health, we found a U-shaped association where both short (<7 h) and long (>8 h) sleep duration were negatively associated with self-reported health for both genders, consistent with prior findings [49–52]. The magnitude of the association was greater for long in comparison with short sleep duration. No significant difference in sleep duration was found between men and women within-countries, but there were cross-national variations (Additional file 2: Table S1). Currently, it is unclear whether older men or older women actually sleep longer on average [53–55]. Nevertheless, both elderly men and women allocate more time to sleep than any other time use activity [6, 24]. This time use pattern is expected among older individuals due to the increasing incidence of adverse health conditions [6]. Conversely, both short and long sleep duration have been found to be associated with adverse health outcomes including diabetes mellitus [55, 56] obesity [57, 58], osteoporosis [59] and hypertension [60]. Furthermore, recent studies suggest that both short and long sleep duration are associated with increased mortality rates [23, 61]. Considering gender, a cohort study by Ikehara et al. [62] showed a U-shaped association between sleep duration and all-cause mortality for both men and women. Even though our data does not permit examining the association with mortality, previous studies have consistently shown a strong relationship between self-reported health and mortality [63, 64].

While the assessment of gender differences in housework and sleep duration with health status was informative, our study further examined the combined associations, as they provide information about a potentially important interactive gender effect. The results showed that the interactive associations between housework, sleep duration and self-reported health vary by gender. Among men, the combination of longest housework hours with either short or long sleep duration yielded a strong positive association with self-reported health. On the contrary, a combination of longest housework hours with either short or long sleep duration yielded a negative association on self-reported health among women. The result suggests that regardless of sleep duration, less housework was associated with poor health status among both genders.

There is no prior evidence of the combined association of sleep duration and time devoted to housework on health status. Nonetheless, Kiosses and Alexopoulos [65] found that older adults who report higher levels of depression devote less time to housework and other instrumental activities of daily living (IADL) such as shopping and meal preparation. This negative association can be explained by little or no physical or mental energy associated with less housework, as physical inactivity has been found to play a significant role in the development of chronic diseases [66]. As discussed above, short and long sleep durations have also been linked to poor health [55, 56], therefore our findings of the combined effects may be attributed to the negative correlations between less housework, poor sleep and health. Regarding long housework hours and sleep duration, we observed a different pattern among both genders (Fig. 1a and b). For men, long hours of housework was associated with good health status regardless of sleep duration, whereas these very long hours of housework combined with either short or long sleep was negatively associated with health among women. In fact, these patterns suggest that long housework hours is less sensitive to elderly men’s health [67] since the impact of long housework and health status appeared to be least influenced by sleep duration.

### Limitations and strengths

Our study has some limitations. First, the cross-sectional design of the study prevents conclusions about causality because the association between sleep duration, housework hours and self-reported health may be reciprocal. Second, this study relied on subjective measures to assess duration of sleep, housework and health. However, time use estimates of daily activities have been found to be more accurate and reliable in time use surveys than survey estimates [68, 69]. Notwithstanding, we acknowledge that sleep disturbance [70], may impinge on sleep quality and duration [71], but sleep quality cannot be assessed with time use surveys [24]. Thus, future research should explore objective and sophisticated time use data collection technologies such as smart-phone apps and actigraphy. Third, due to data availability and constrains, we used diary data of time use surveys that have been collected at different points in time with different modes of data collection in the chosen countries, but evaluation studies suggest that these differences do not affect the comparability of the data [11]. Despite these limitations, this current study provides an initial overview of housework activities, sleep duration and their correlations with self-reported health of the population aged 65+ years using a large-scale, homogeneous and comparative set of time use data in Europe as well as the US.

## Conclusions

We provide the first evidence of the associations between housework activities, sleep duration and self-reported health among older individuals in selected high-income countries. Our findings suggest that housework activities remain strongly gendered even after retirement. Our findings further suggest that even though time allocation to housework activities may be beneficial to the health among elderly men and women, women have higher odds of reporting poor health when more time is devoted total housework combined with either short or long sleep duration.

## Additional files

Additional file 1: Table S2. General description of total housework and sleep hours ( means and SD), men and women, by country. (DOCX 15 kb)

Additional file 2: Table S1. Typology of activities. (DOCX 16 kb)
