# Supplementary material for: Investigating the associations between productive housework activities, sleep hours and self-reported health among elderly men and women in western industrialised countries
Source: BMC Public Health. 2018 Jan 11;18:110. doi: 10.1186/s12889-017-4979-z (PMC5763579; doi:10.1186/s12889-017-4979-z)
Supplement: Supplementary file 1 — General description of total housework and sleep hours ( means and SD), men and women, by country. (DOCX 15 kb) [file 12889_2017_4979_MOESM1_ESM.docx]

| **S2 Table**.General description of total housework and sleep hours ( means and SD), men and women, by country | | | | | | |
| --- | --- | --- | --- | --- | --- | --- |
|  | **Total housework hours/day** | | **Sleep hours/day** | |  |  |
|  | Mean | SD | Mean | SD |  |  |
| **Men** |  |  |  |  |  |  |
| Germany | 4.22 | 2.61 | 8.69 | 1.55 |  |  |
| Italy | 2.73 | 2.50 | 9.41 | 1.86 |  |  |
| Spain | 2.49 | 2.54 | 10.11 | 2.24 |  |  |
| UK | 3.94 | 2.42 | 8.59 | 1.66 |  |  |
| USA | 3.17 | 2.89 | 9.04 | 2.18 |  |  |
| Netherlands | 4.09 | 2.72 | 9.02 | 1.51 |  |  |
| France | 3.20 | 2.32 | 9.94 | 2.22 |  |  |
| **Women** |  |  |  |  |  |  |
| Germany | 5.12 | 2.43 | 8.69 | 1.68 |  |  |
| Italy | 5.16 | 2.74 | 9.40 | 1.93 |  |  |
| Spain | 4.80 | 2.72 | 9.82 | 2.19 |  |  |
| UK | 4.60 | 2.33 | 8.62 | 1.75 |  |  |
| USA | 4.03 | 3.00 | 8.97 | 2.11 |  |  |
| Netherlands | 4.69 | 2.42 | 9.12 | 1.93 |  |  |
| France | 4.55 | 2.29 | 10.01 | 2.39 |  |  |
