# Supplementary material for: Investigating the associations between productive housework activities, sleep hours and self-reported health among elderly men and women in western industrialised countries
Source: BMC Public Health. 2018 Jan 11;18:110. doi: 10.1186/s12889-017-4979-z (PMC5763579; doi:10.1186/s12889-017-4979-z)
Supplement: Supplementary file 2 — Typology of activities. (DOCX 16 kb) [file 12889_2017_4979_MOESM2_ESM.docx]

| **S1** **Table.** Typology of activities | |  |  |  |
| --- | --- | --- | --- | --- |
| **Broad categories of activity** | | **Name of variable** **(harmonised)** | | **Description** |
| **1. Paid work** |  | AV01 |  | Paid work |
|  |  | AV02 |  | Paid work at home |
|  |  | AV03 |  | Second job |
|  |  | AV05 |  | Travel to/ from work |
|  |  |  |  |  |
| **2.Housework** |  | AV06 |  | Cooking/Washing up |
|  |  | AV07 |  | Housework |
|  |  | AV08 |  | Odd jobs |
|  |  | AV09 |  | Gardening, pets |
|  |  | AV10 |  | Shopping |
|  |  | AV11 |  | Child care |
|  |  | AV12 |  | Domestic travel |
|  |  |  |  |  |
| **3.Active leisure** |  | AV23 |  | Civic duties |
|  |  | AV19 |  | Active sport |
|  |  | AV21 |  | Walks |
|  |  | AV17 |  | Leisure travel |
|  |  | AV18 |  | Excursions |
|  |  | AV22 |  | Religious activities |
|  |  | AV24 |  | Cinema, theatre |
|  |  | AV26 |  | Social club |
|  |  | AV27 |  | Pub |
|  |  | AV28 |  | Restaurant |
|  |  | AV29 |  | Visiting friends |
|  |  | AV04 |  | School/classes |
|  |  | AV20 |  | Passive/observer sports |
|  |  | AV33 |  | Study |
|  |  | AV34 |  | Reading books |
|  |  | AV35 |  | Reading papers and magazines |
|  |  | AV37 |  | Conversation |
|  |  | AV38 |  | Entertaining friends |
|  |  | AV39 |  | Knitting sewing etc. |
|  |  | AV40 |  | Other hobbies |
|  |  |  |  |  |
| **4.Passive leisure** |  | AV30 |  | Listening to radio |
|  |  | AV31 |  | Television, video |
|  |  | AV32 |  | Listening to tapes etc. |
|  |  | AV36 |  | Relaxing |
|  |  |  |  |  |
| **5.Personal activity** |  | AV13 |  | Dressing/toilet |
|  |  | AV14 |  | Personal Services |
|  |  | AV15 |  | Meals, snacks |
|  |  | AV16 |  | Sleep |
